# Finding a predictive index for evaluating the efficacy of neoadjuvant therapy in locally advanced gastric cancer patients

**DOI:** 10.1186/s12957-026-04373-9

**Published:** 2026-05-25

**Authors:** Peijun Wang, Gaofeng Huang, Jindai Zhang, Qiang Fu

**Affiliations:** General Surgery, Affiliated Cancer Hospital of Zhengzhou University, Henan Cancer Hospital, No. 127 Dongming Road, Zhengzhou, Henan Province China

**Keywords:** Neoadjuvant immuno-chemotherapy, Tumor regression grade, Clinical predictive pathological remission score, Predictive indicator, Locally advanced gastric cancer

## Abstract

**Background:**

Emerging evidence suggests that the addition of immunotherapy may improve tumor regression grade (TRG) in locally advanced gastric cancer (LAGC) patients. However, the clinical benefit of immunotherapy in patients with low PD-1 expression remains a subject of debate and requires further exploration. Importantly, a proportion of Neoadjuvant immuno-chemotherapy group (NICT) patients did not present significant benefits from neoadjuvant therapy. The aim and objectives of our study is to stratify LAGC patients who can benefit from neoadjuvant therapy based on TRG results.

**Methods:**

We conducted a retrospective study (2020–2023) included 94 LAGC patients (cT3N + M0, 4a-bNanyM0) with gastric adenocarcinoma who receiving neoadjuvant therapy (57 patients in the neoadjuvant chemotherapy (NCT) group, and 37 in NICT group). All patients received ulterior radical surgery, and clinicopathological data were retrospectively reviewed and analyzed. We introduced multivariate analysis demonstrated significant associations between TRG and Tumor differentiation (OR, 7.985; 95% CI, 0.998–34.370; *p* = 0.007), N stage (OR, 13.286; 95% CI, 1.861–50.324; *p* = 0.013), tumor size (OR, 5.397; 95% CI, 1.174–24.818; *p* = 0.030), and immunotherapy (OR, 16.872; 95% CI, 2.889–75.563; *p* < 0.001). We developed the Clinical Predictive Pathological Remission Score (CPPRS) according to the four indicators mentioned above, assigning 0 points for immunotherapy, well-differentiated tumors, absence of lymph node metastasis, and tumor size < 3.55 cm, while 1 point was assigned for NCT, moderate/poor differentiation, tumor size > 3.55 cm, and lymph node metastasis.

**Results:**

NICT group exhibited a significantly higher TRG0 rate compared to the NCT group, independent of PD-1 expression (9/37 vs. 2/57, p = 0.004). Tumor differentiation (OR, 7.985; 95% CI, 0.998–34.370; *p* = 0.007), N stage (OR, 13.286; 95% CI, 1.861–50.324; *p* = 0.013), tumor size (OR, 5.397; 95% CI, 1.174–24.818; *p* = 0.030), and immunotherapy (OR, 16.872; 95% CI, 2.889–75.563;* p* < 0.001) were significantly associated with TRG. CPPRS demonstrated a strong correlation with TRG (*p* < 0.001).

**Conclusions:**

The addition of immunotherapy improves TRG in LAGC patients without the need for PD-1 expression assessment. CPPRS serves as a valuable predictive tool for evaluating the efficacy of neoadjuvant therapy in LAGC patients.

**Supplementary Information:**

The online version contains supplementary material available at 10.1186/s12957-026-04373-9.

## Introduction

Gastric cancer represents a significant global health burden, ranking as the third most prevalent malignancy and the third leading cause of cancer-related mortality worldwide, with particularly high incidence rates in China [1–3]. Locally advanced gastric cancer (LAGC) constitutes approximately 60% of all gastric cancer cases. While surgical intervention remains the cornerstone of radical treatment for gastric cancer, the conventional approach of radical surgery combined with postoperative adjuvant therapy has reached a therapeutic plateau due to persistent challenges of micrometastasis [4–6]. Over the past decade, clinical evidence has consistently demonstrated that perioperative chemotherapy significantly enhances R0 resection rates and improves overall survival (OS) compared to surgery alone [7, 8]. Neoadjuvant therapy has shown particular promises in tumor downstaging, elimination of micrometastasis, and extension of disease-free survival (DFS). However, the OS and DFS in these patients still need to be further improved. [9, 10].

The emergence of immunotherapy has revolutionized anticancer treatment strategies by targeting tumor immune evasion mechanisms through immune activation and pathway modulation [11, 12]. Current treatment guidelines, including the National Comprehensive Cancer Network (NCCN) Clinical Practice Guidelines for Gastric Cancer (1st edition, 2021), recommend neoadjuvant chemotherapy for patients with good performance status, potentially resectable lesions, and clinical staging of cT2 ~ 4N0 ~ 3. The European Society of Oncology (ESMO) endorses platinum/fluorouracil combination therapy as the preferred perioperative treatment for LAGC, based on findings from the MAGIC and FNCLCC/FFCD clinical trials [13, 14]. In the evolving landscape of cancer therapy, immunotherapy has demonstrated superior tumor reduction efficacy and pathologic remission [15–17]. Notably, the combination of immunomonoclonal antibodies with the platinum plus 5-fluorouracil drugs has shown significant improvement in pathological complete response (pCR) rates in LAGC patients [18]. However, we found that some patients with LAGC did not achieve better TRG outcomes even with high CPS scores, in contrast, some patients with low CPS scores achieved better TRG outcomes. The observed heterogeneity in immunotherapy response among LAGC patients underscores the critical need to identify predictive clinical biomarkers to optimize patient selection for immunotherapeutic interventions.

## Materials and methods

### Patients

This retrospective study analyzed data from patients with locally advanced gastric cancer (LAGC) who underwent neoadjuvant therapy followed by radical gastrectomy at the Affiliated Cancer Hospital of Zhengzhou University between January 2020 and June 2023. The patients included in this study ranged in age from 32 to 73 years. Inclusion criteria comprised all patients with cT3N + M0 or 4a-bNanyM0 gastric adenocarcinoma cancer(ICD-10; C16) which underwent a pathological biopsy, as confirmed by computed tomography (CT) imaging, Her2-negative status determined by immunohistochemistry, Eastern Cooperative Oncology Group performance status of 0–1, and completion of 2–3 cycles of SOX regimen (NCT group) or it combined with immunotherapy (NICT group). Exclusion criteria included presence of distant metastasis, gastric stump cancer, concurrent malignancies, intolerance to neoadjuvant therapy, or prior receipt of targeted therapy or chemoradiotherapy.

### Treatments

All patients underwent multidisciplinary team (MDT) evaluation prior to treatment initiation. The NACT group received 2–3 cycles of SOX regimen (oxaliplatin: 130 mg/m2 intravenously on day 1 every 3 weeks; S1: 40 mg/m2 orally twice daily for 2 weeks every 3 weeks), while the NICT group received combined with immunotherapy (Sintilimab, Navulizumab, and Treprizumab). As a retrospective clinical study, the immunotherapy or patient assignment to each group was based on usual clinical practice. Pre-treatment assessments included comprehensive physical examination and hematological evaluation before each cycle, with additional monitoring of corticosteroid levels, thyroid function, and myocardial enzymes for NICT patients. Disease status was evaluated by CT imaging following every 2–3 treatment cycles. Radical resection with R0 resection was performed in LAGC patients who achieve tumor regression or no significant progression on contrast-enhanced CT compared with baseline. The same criteria were applied in both the NACT and NAICT groups. The postoperative pathological TNM staging determined according to tumor regression grade (TRG) criteria. TRG 0 indicates no viable tumor cells (complete response); TRG 1 indicates single cells or small clusters of tumor cells remaining (partial response); TRG 2 indicates residual tumor cells greater than fibrosis (minimal response); TRG 3 indicates very little or no tumor cell death or extensive residual cancer cells (poor response).

### Statistical analysis

Statistical analyses were performed using SPSS software (version 25.0, IBM Corp., Armonk, NY). We assessed the normality of the variables using the Kolmogorov‑Smirnov test (K‑S test), continuous variables were expressed as mean ± standard deviation for normally distributed data (as tumor markers, tumor size, et al.), while categorical variables were presented as frequencies (percentages). Comparative analyses of proportions were conducted using chi-square or Fisher's exact tests, while mean differences were assessed using Student's t-test or Mann–Whitney U test, as appropriate. Univariate and multivariate analyses were performed using Cox proportional hazards regression models, with risk factors quantified by odds ratio (OR) and corresponding 95% confidence intervals (CI). Two-tailed *p*-values < 0.05 were considered statistically significant.

## Results

The study cohort comprised 94 patients with locally advanced gastric cancer (LAGC) who initiated perioperative chemotherapy, with a median age of 59.86 years. Among these patients, 57 patients (60.64%) received neoadjuvant chemotherapy (NCT) based on platinum and fluorouracil regimens, while 37 patients (39.36%) were enrolled in the neoadjuvant immunotherapy combined with chemotherapy (NICT) group. All patients demonstrated adequate tolerance to preoperative treatment, with no statistically significant differences in adverse events observed between the two treatment groups (p = 0.623).

The NCT group consisted of 48 male patients (51.1%) and 9 female patients (9.6%), with clinical tumor staging (cT) distribution of 16 patients (17.0%) at cT3 and 41 patients (43.6%) at cT4. The nodal staging (cN) distribution included 31 patients (33.0%) at cN0-1 and 26 patients (27.6%) at cN2-3. Regarding TNM staging, 16 patients (17.0%) were classified as stage II and 41 patients (43.6%) as stage III. The NAICT group comprised 28 male patients (29.8%) and 9 female patients (9.6%), with cT staging distribution of 17 patients (18.1%) at cT3 and 20 patients (21.3%) at cT4. The cN staging included 24 patients (26.6%) at cN0-1 and 13 patients (14.9%) at cN2-3, while TNM staging consisted of 16 patients (17.0%) at stage II and 21 patients (22.3%) at stage III. No significant differences were observed between the groups in combined positive score (CPS) which is an indicator used to assess the expression level of PD-L1 protein in gastric cancer tissue. Tumor‑associated markers including CA724, CA199, CEA, hemoglobin, and the neutrophil‑to‑lymphocyte ratio (NLR) have been widely reported linked to tumor recurrence and metastasis. However, whether these markers are associated with tumor regression grade (TRG) remains unclear. Accordingly, we performed the present study to explore this potential correlation (Table 1, 2).

**Table 1:** Baseline characteristics of patients according to the treatment plan

Patient characteristic	NICT group (*n* = 37)	NCT group (*N* = 57)	*P value*
Age (years)	59.97 ± 9.12	59.79 ± 8.83	0.923
Gender, *n* (%)			0.223
Male	28(29.8%)	48(51.1%)	
Female	9(9.6%)	9(9.6%)	
Primary tumour location, *n* (%)			0.08
Upper	22(23.4%)	46(48.9%)	
Middle	8(8.5%)	6(6.4%)	
Lower	7(7.4%)	5(5.3%)	
Tumour differentiation, *n* (%)			0.571
Well differentiated	10(10.6%)	19(20.2%)	
Moderately differentiated	21(22.3%)	26(27.7%)	
Poorly differentiated	6(6.4%)	12(12.8%)	
T stage			0.083
cT3	17(18.1%)	16(16.0%)	
cT4	20(21.3%)	41(43.6%)	
N stage			0.075
cN0	9(9.6%)	4(4.3%)	
cN1	15(16.0%)	27(28.7%)	
cN2	9(9.6%)	13(13.8%)	
cN3	4(4.3%)	13(13.8%)	
TNM stage			0.338
IIb	16(17.0%)	17(18.1%)	
IIIa	11(11.7%)	17(18.1%)	
IIIb	6(6.4%)	9(9.6%)	
IIIc	4(4.3%)	14(14.9%)	
Preoperative PD-L1 expression, *n* (%)			0.177
CPS < 5	9(22.0%)	10(24.4%)	
CPS ≥ 5	15(36.6%)	7(17.1%)	
Tumor size	3.55 ± 1.85	3.96 ± 2.28	0.375
Type of gastrectomy, *n* (%)			0.026
Distal Partial	7(7.4%)	2(2.1%)	
Whole	30(31.9%)	55(58.5%)	
Nerve invasion, *n* (%)			0.001
Positive	7(7.6%)	26(28.3%)	
Negative	30(33.0%)	29(31.5%)	
Unknown	1(1.1%)	2(2.1%)	
Vessel invasion, *n* (%)			0.011
Positive	14(15.2%)	36(39.1%)	
Negative	23(25.0%)	19(20.7%)	
Tumor markers before treatment			
CEA(ng/mL)	4.48 ± 5.48	26.11 ± 131.96	0.329
CA199(U/mL)	74.53 ± 186.66	81.35 ± 175.33	0.859
CA724(U/mL)	21.81 ± 62.69	8.61 ± 17.36	0.136
Pre-NLR	2.16 ± 0.95	2.17 ± 0.84	0.952
Pre-treatment Hb	120.30 ± 24.52	124.21 ± 23.66	0.442
Tumor markers after treatment			
CEA(ng/mL)	3.95 ± 4.74	9.21 ± 29.50	0.286
CA199(U/mL)	45.88 ± 128.99	70.16 ± 171.81	0.464
CA724(U/mL)	11.57 ± 27.39	9.33 ± 22.77	0.669
Preoperative NLR	1.96 ± 0.99	1.70 ± 0.95	0.202
Preoperative Hb	118.19 ± 18.41	121.05 ± 16.68	0.437
ypT stage			0.009
T0	9(9.6%)	2(2.2%)	
T1	2(2.1%)	3(3.3%)	
T2	6(6.4%)	5(5.4%)	
T3	10(10.6%)	15(16.3%)	
T4	10(10.6%)	32(34.0%)	
ypN stage			0.056
N0	20(21.3%)	21(22.3%)	
N1	6(6.4%)	12(12.8%)	
N2	9(9.6%)	12(12.8%)	
N3	2(2.1%)	12(12.8%)	
ypTNM stage			0.004
0	9(9.6%)	2(2.1%)	
Ia	1(1.1%)	3(3.3%)	
Ib	5(5.3%)	1(1.1%)	
IIa	5(5.3%)	11(12.2%)	
IIb	5(5.3%)	13(13.8%)	
IIIa	7(7.8%)	6(6.4%)	
IIIb	3(3.2%)	9(10.0%)	
IIIc	2(2.1%)	12(12.8%)	
TRG			0.004
0	9(9.6%)	2(2.1%)	
1	7(7.4%)	9(9.6%)	
2	16(17.0%)	24(25.5%)	
3	5(5.3%)	22(23.4%)	
Nerve invasion, *n* (%)			0.001
Positive	7(7.6%)	26(28.3%)	
Negative	30(33.0%)	29(31.5%)	
Unknown	1(1.1%)	2(2.1%)	
Vessel invasion, *n* (%)			0.011
Positive	14(15.2%)	36(39.1%)	
Negative	23(25.0%)	19(20.7%)

**Table 2:** The characteristics of patients according to the TRG

Patient characteristic	TRG 0 (*n* = 11)	TRG 1 (*n* = 16)	TRG 2 (*n* = 40)	TRG 3 (*n* = 27)	*P value*
Age (years)	61.18 ± 6.24	59.94 ± 6.81	57.93 ± 9.86	62.15 ± 9.15	0.273
Gender, *n* (%)					0.652
Male	10(10.6%)	14(14.9%)	31(33.0%)	21(22.3%)	
Female	1(1.1%)	2(2.1%)	9(9.6%)	6(6.4%)	
Primary tumour location, *n* (%)					0.350
Upper	7(7.4%)	10(10.6%)	30(31.9%)	21(22.3%)	
Middle	1(1.1%)	2(2.1%)	6(6.4%)	5(5.3%)	
Lower	3(3.2%)	4(4.3%)	4(4.3%)	1(1.1%)	
Tumour differentiation, *n* (%)					0.007
Well differentiated	7(7.4%)	8(8.5%)	8(8.5%)	6(6.4%)	
Moderately differentiated	4(4.3%)	8(8.5%)	19(20.2%)	16(17.0%)	
Poorly differentiated	0(0.0%)	0(0.0%)	13(13.8%)	5(5.3%)	
T stage					0.007
cT3	6(6.4%)	10(10.6%)	13(13.8%)	4(4.3%)	
cT4	5(5.3%)	6(6.4%)	27(28.7%)	23(24.5%)	
N stage					< 0.001
cN0	5(5.3%)	2(2.1%)	1(1.1%)	4(4.3%)	
cN1	6(6.4%)	12(12.8%)	19(20.2%)	6(6.4%)	
cN2	0(0.0%)	2(2.1%)	11(11.7%)	9(9.6%)	
cN3	0(0.0%)	0(0.0%)	9(9.6%)	8(8.5%)	
TNM stage					0.002
IIb	9(9.6%)	7(7.4%)	9(9.6%)	8(8.5%)	
IIIa	2(2.1%)	8(8.5%)	14(14.9%)	4(4.3%)	
IIIb	0(0.0%)	1(1.1%)	8(8.5%)	6(6.4%)	
IIIc	0(0.0%)	0(0.0%)	9(9.6%)	9(9.6%)	
Preoperative PD-L1 expression in NAICT, *n* (%)					0.327
CPS < 5	1(2.4%)	4(9.8%)	11(26.8%)	3(7.3%)	
CPS ≥ 5	4(9.8%)	7(17.1%)	7(17.1%)	4(9.8%)	
Type of gastrectomy, *n* (%)					0.008
Distal Partial	3(3.2%)	4(4.3%)	1(1.1%)	1(1.1%)	
Whole	8(8.5%)	12(12.8%)	39(41.5%)	26(27.7%)	
Nerve invasion, *n* (%)					0.003
Positive	0(0.0%)	3(3.3%)	19(20.7%)	14(15.2%)	
Negative	11(12.0%)	13(14.3%)	21(22.8%)	11(12.0%)	
Vessel invasion, *n* (%)					< 0.001
Positive	0(0.0%)	5(5.4%)	25(27.2%)	20(21.7%)	
Negative	11(12.0%)	11(12.0%)	15(16.3%)	5(5.4%)	
Tumor markers before treatment					
CEA(ng/mL)	3.57 ± 4.23	5.70 ± 7.87	34.22 ± 159.38	6.83 ± 8.27	0.642
CA199(U/mL)	23.62 ± 35.20	32.07 ± 31.07	91.45 ± 204.96	110.40 ± 216.37	0.370
CA724(U/mL)	12.14 ± 29.30	16.69 ± 56.86	15.97 ± 48.99	9.34 ± 18.72	0.919
Pre-NLR	2.13 ± 0.85	2.02 ± 0.81	2.02 ± 0.78	2.48 ± 1.03	0.163
Pre-treatment Hb	128.45 ± 25.26	121.88 ± 16.91	128.05 ± 23.09	112.81 ± 26.09	0.062
Tumor markers after treatment					
CEA(ng/mL)	4.66 ± 2.44	3.18 ± 1.87	10.56 ± 5.57	5.42 ± 3.01	0.665
CA199(U/mL)	12.27 ± 7.98	24.07 ± 6.92	45.66 ± 17.41	124.08 ± 47.98	0.077
CA724(U/mL)	1.98 ± 0.49	8.23 ± 4.40	13.98 ± 5.14	9.16 ± 3.55	0.046
Preoperative NLR	1.78 ± 0.93	2.14 ± 0.32	1.64 ± 0.13	1.85 ± 0.20	0.365
Preoperative Hb	120.55 ± 17.72	115.56 ± 17.93	122.28 ± 16.94	118.78 ± 17.82	0.605
Tumor size	2.60 ± 1.13	3.22 ± 1.73	3.55 ± 1.87	5.03 ± 2.12	0.002
ypT stage					< 0.001
T0	11(12.0%)	0(0.0%)	0(0.0%)	0(0.0%)	
T1	0(0.0%)	4(4.3%)	1(1.1%)	0(0.0%)	
T2	0(0.0%)	4(4.3%)	4(4.3%)	3(3.2%)	
T3	0(0.0%)	5(5.3%)	15(16.0%)	5(5.3%)	
T4	0(0.0%)	3(3.2%)	20(21.7%)	19(20.2%)	
ypN stage					< 0.001
N0	11(12.2%)	10(10.6%)	16(17.0%)	4(4.3%)	
N1	0(0.0%)	4(4.3%)	7(7.4%)	7(7.4%)	
N2	0(0.0%)	2(2.1%)	11(11.7%)	8(8.5%)	
N3	0(0.0%)	0(0.0%)	6(6.4%)	8(8.5%)	
ypTNM stage					< 0.001
0	11(11.7%)	0(0.0%)	0(0.0%)	0(0.0%)	
Ia	0(0.0%)	3(3.2%)	1(1.1%)	0(0.0%)	
Ib	0(0.0%)	2(2.1%)	3(3.2%)	1(1.1%)	
IIa	0(0.0%)	6 (6.4%)	7(7.4%)	3(3.2%)	
IIb	0(0.0%)	4(4.3%)	9(9.6%)	5(5.3%)	
IIIa	0(0.0%)	0(0.0%)	9(9.6%)	4(4.3%)	
IIIb	0(0.0%)	1(1.1%)	6(6.4%)	5(5.3%)	
IIIc	0(0.0%)	0(0.0%)	5(5.3%)	9(9.6%)	
Treatment grouping					0.004
NICT	9 (9.6%)	7(7.4%)	16 (17.0%)	5(5.3%)	
NCT	2(2.1%)	9(9.6%)	24 (25.5%)	22 (23.4%)	

### Response evaluation by downstaging

Tumor size measurements revealed no significant difference between the NCT and NICT groups (3.96 ± 2.28 cm vs. 3.55 ± 1.85 cm, *p* = 0.375). The addition of immunotherapy demonstrated enhanced downstaging efficacy, particularly in T staging and TNM staging. Among the cohort, 68 patients (72.34%) achieved downstaging, 29 patients (27.66%) maintained stable disease, and no patients experienced disease upstaging. Histopathological complete response was observed in 11 patients (11.70%). Comparative analysis revealed that the NICT group achieved significantly better tumor regression grade (TRG) outcomes compared to the NCT group (*p* = 0.004) (Table 1).

### Postoperative pathological information

To comprehensively assess the role of CPS in LAGC patients, we incorporated TRG outcomes for secondary analysis. Our findings revealed that NICT group patients exhibited a significantly higher pathological complete response (pCR) rate compared to the NCT group (24.32% vs. 3.51%, p < 0.001). TRG distribution demonstrated significant differences between groups, with TRG 0, 1, 2, and 3 observed in 2 (2.1%), 9 (9.6%), 23 (24.5%), and 22 (23.4%) patients in the NCT group, compared to 9 (9.6%), 7 (7.4%), 16 (17.0%), and 5 (5.3%) patients in the NAICT group, respectively (*p* = 0.004). Multivariate analysis identified significant associations between TRG and tumor differentiation, tumor size, clinical N stage (cN), clinical TNM stage (cTNM), and immunotherapy administration (Table 3). Notably, tumor size demonstrated an inverse correlation with TRG 0 probability (*p* = 0.002). While no statistically significant association was observed between TRG and CPS, potentially due to the limited NICT cohort size, NICT patients achieved higher TRG 0 rates irrespective of CPS scores (*p* = 0.001) (Table 2). Furthermore, pretreatment tumor markers and hemoglobin levels showed no significant correlation with TRG outcomes.

**Table 3:** Cox regression analysis of predictors for TRG in LAGC patients

Factors	TRG					
	Univariate analysis			Multivariate analysis		
	OR	95%CI	*P* value	OR	95%CI	*P* value
Gender, (Male/Female)	1.395	0.778–2.499	0.264			
Tumor size(cm)						
> 3.55 vs < 3.55	1.953	1.198–3.185	0.007	5.397	1.174–24.818	0.030
Tumour differentiation, *n* (%)				7.985	0.998–34.370	0.007
Well vs Moderately and Poorly	2.011	1.239–3.266	0.004			
T stage				1.205	0.079–18.284	0.893
cT3 vs cT4	1.885	1.308–2.444	0.021			
N stage				13.286	1.861–50.324	0.013
cN0-1vs N2-3	3.386	1.825–6.281	< 0.001			
TNM stage				5.375	0.499–57.808	0.167
IIb IIIa vs IIIb IIIc	2.874	1.366–4.448	0.012			
Type of gastrectomy, *n* (%)						
Distal Partial vs Whole	1.357	1.136–1.797	0.069			
Treatment grouping				16.872	2.889–75.563	< 0.001
NICT vs NCT	2.272	1.389–3.717	0.001			

Our analysis confirmed the association between immunotherapy and TRG in LAGC patients, identifying several TRG-related prognostic factors. Univariate logistic regression analysis revealed significant associations between TRG and tumor size (OR, 1.953; 95%CI, 1.198–3.185; *p* = 0.007), tumor differentiation (OR, 2.011; 95%CI, 1.239–3.266; *p* = 0.004), T stage (OR, 1.885; 95%CI, 1.308–2.444; *p* = 0.021), N stage (OR, 3.386; 95%CI, 1.825–6.281; *p* < 0.001), TNM stage (OR, 2.874; 95%CI, 1.366–4.448; *p* = 0.012), and immunotherapy (OR, 8.621; 95%CI, 2.270–52.756; *p* = 0.006) (Table 3). Multivariate analysis further substantiated these findings, demonstrating significant associations between TRG and Tumor differentiation (OR, 7.985; 95% CI, 0.998–34.370; *p* = 0.007), N stage (OR, 13.286; 95% CI, 1.861–50.324; *p* = 0.013), tumor size (OR, 5.397; 95% CI, 1.174–24.818; *p* = 0.030), and immunotherapy (OR, 16.872; 95% CI, 2.889–75.563;* p* < 0.001) (Table 3).

### Clinical predictive pathological remission score(CPPRS)

Our study demonstrated a significant association between immune therapy and TRG in LAGC patients, with additional correlations observed between TRG and tumor differentiation, preoperative lymph node metastasis and tumor size. To enhance the predictive accuracy of preoperative neoadjuvant therapy efficacy, we developed the Clinical Predictive Pathological Remission Score (CPPRS). Initially, we determined the optimal cutoff value for tumor size to be 3.55 cm for TRG 0 by the AUC analysis in SPSS, and adding a figure with AUC analysis (*p* = 0.046; 0.244–0.470) in the supplementary Fig. 1. Subsequently, we employed a dichotomous scoring approach, assigning 0 points to neoadjuvant immune-chemotherapy (NICT), well-differentiated tumors, absence of lymph node metastasis, and tumor size < 3.55 cm (Supplementary Fig. 1). Conversely, 1 point was assigned to neoadjuvant chemotherapy (NCT), moderately and poorly differentiated tumors, presence of lymph node metastasis and tumor size ≥ 3.55 cm. Our findings revealed a strong correlation between CPPRS and TRG (*p* < 0.001), with lower CPPRS scores indicative of superior pathological remission (Table 4). Furthermore, we validated CPPRS as a robust predictor of TRG in LAGC patients following neoadjuvant therapy, as evidenced by the area under the curve (AUC) analysis for TRG (*p* < 0.001) (Supplementary Fig. 2).

**Table 4:** The efficacy of CPPRS for TRG in LAGC patients

CPPRS	TRG					*P*-value
	0	1	*2*	3	Total	< 0.001
0	6(100.0%)	0(0.0%)	0(0.0%)	0(0.0%)	6	
1	3(16.7%)	6(33.3%)	7(38.9%)	2(11.1%)	18	
2	2(7.4%)	7(25.9%)	14(51.9%)	4(14.8%)	27	
3	0(0.0%)	3(11.5%)	11(42.3%)	12(46.2%)	26	
4	0(0.0%)	0(0.0%)	8(47.1%)	9(46.2%)	17	

## Discussion

The 5-year survival rates for LAGC patients remain suboptimal, with reported rates of 30.5%, 20.1%, and 8.3% for stages IIIA, IIIB, and IIIC, respectively [19]. While neoadjuvant chemotherapy (NCT) has been extensively employed over the past decade to enhance R0 resection rates and disease-free survival (DFS), its clinical outcomes have been limited by low rates of complete pathological remission. Recent advancements in immunotherapy have demonstrated promising efficacy in advanced gastric cancer [20], however, the therapeutic implications of programmed death-1 (PD-1) expression levels in LAGC patients remain a subject of ongoing debate [21, 22], particularly in cases with low PD-1 expression, warranting further investigation.

TRG serves as a well-established metric for assessing the efficacy of neoadjuvant therapies. Our findings corroborate previous studies indicating that neoadjuvant immunochemotherapy yields superior TRG outcomes compared to NCT in LAGC patients [23]. Notably, our study revealed that the addition of immunotherapy significantly increased the probability of achieving TRG0, irrespective of PD-1 expression status. Through logistic regression analysis, we identified immunotherapy as a robust predictor of TRG in LAGC patients, suggesting that even those with low PD-1 expression may derive therapeutic benefit from immunotherapy.

Consistent with emerging evidence [24], our results support the prognostic significance of TRG, as it has been strongly associated with survival outcomes. Importantly, multivariate logistic regression analysis identified NICT as an independent predictor of TRG (OR16.872, 95% CI 2.889–75.563, p < 0.001). These findings suggest that the incorporation of immunotherapy may confer survival benefits to LAGC patients, including those with low PD-1 expression. However, our study did not identify a significant association between combined positive score (CPS ≥ 5 or < 5) and TRG, potentially due to limited sample size, highlighting the need for further investigation.

In conclusion, our study demonstrates that the addition of immunotherapy to LAGC treatment regimens can enhance therapeutic efficacy, independent of PD-1 expression status, thereby offering a promising therapeutic strategy for this patient population.

Numerous investigations have examined the prognostic significance of diverse serum tumor markers in LAGC patients undergoing neoadjuvant chemotherapy. Prior studies have suggested that a reduction in CA724 levels may correlate with improved prognosis and serve as an indicator of neoadjuvant chemotherapy efficacy [25]. However, the potential association between serum tumor markers and TRG in patients receiving neoadjuvant therapy remains insufficiently explored. In the present study, pretreatment levels of CA199, CEA, and CA724 were not predictive of TRG.

Our study revealed that not all patients with LAGC undergoing neoadjuvant immunotherapy combined with chemotherapy achieved favorable TRG, indicating that a subset of patients obtained limited benefit from this therapeutic approach. Identifying the relationship between TRG and pretreatment clinical factors could facilitate the stratification of patients unlikely to benefit from neoadjuvant immunotherapy, thereby guiding the selection of alternative treatment strategies, such as upfront surgery or radiotherapy. Through multivariate regression analysis, we identified pretreatment tumor size, tumor differentiation, and lymph node metastasis as significant predictors of TRG. Specifically, tumor size exhibited a positive correlation with tumor burden, which may impair drug permeability and compromise the efficacy of neoadjuvant therapy, consistent with prior findings in LAGC patients receiving neoadjuvant chemotherapy. Furthermore, our results corroborated the findings of Zhu et al., demonstrating a strong association between tumor differentiation and TRG in LAGC patients treated with NICT [26]. Additionally, pretreatment lymph node status, which reflects metastatic potential and chemoresistance, emerged as a predictive factor for TRG, although further investigation is required to validate its role in this context.

In this study, we first demonstrated that the addition of immunotherapy to NICT improved therapeutic outcomes in LAGC patients, independent of PD1 expression. However, a subset of patients still exhibited suboptimal responses to NAICT. To enhance the predictive accuracy of TRG, we developed the CPPRS (Clinical-Pathological Predictive Regression Score), which effectively stratified patients based on their likelihood of achieving favorable TRG. Notably, all patients with a CPPRS of 0 achieved TRG0, while higher CPPRS scores were associated with poorer TRG (p < 0.001).

This study has several limitations, primarily its retrospective design and relatively small sample size, which may introduce selection bias and increase the risk of false-positive or false-negative results. Additionally, the inclusion of patients treated with three distinct immunomodulatory agents (Xindilizumab, Navulizumab, and Treprizumab) may have introduced heterogeneity in the treatment results. Finally, the use of the TRG-Mandard system as the pathological tumor regression grading criterion for gastric cancer remains a subject of debate, potentially limiting the generalizability of our findings, and the CPPRS score still requires multicenter clinical validation.

## Supplementary Information

Supplementary Material 1

## Data Availability

The data that supports the findings of this study are available from the corresponding author upon reasonable request.
