# Supplementary material for: Finding a predictive index for evaluating the efficacy of neoadjuvant therapy in locally advanced gastric cancer patients
Source: World J Surg Oncol. 2026 May 25;24:303. doi: 10.1186/s12957-026-04373-9 (PMC13383457; doi:10.1186/s12957-026-04373-9)

Supplementary Figure 1 The AUC of tumor size for TRG in LAGC patients.


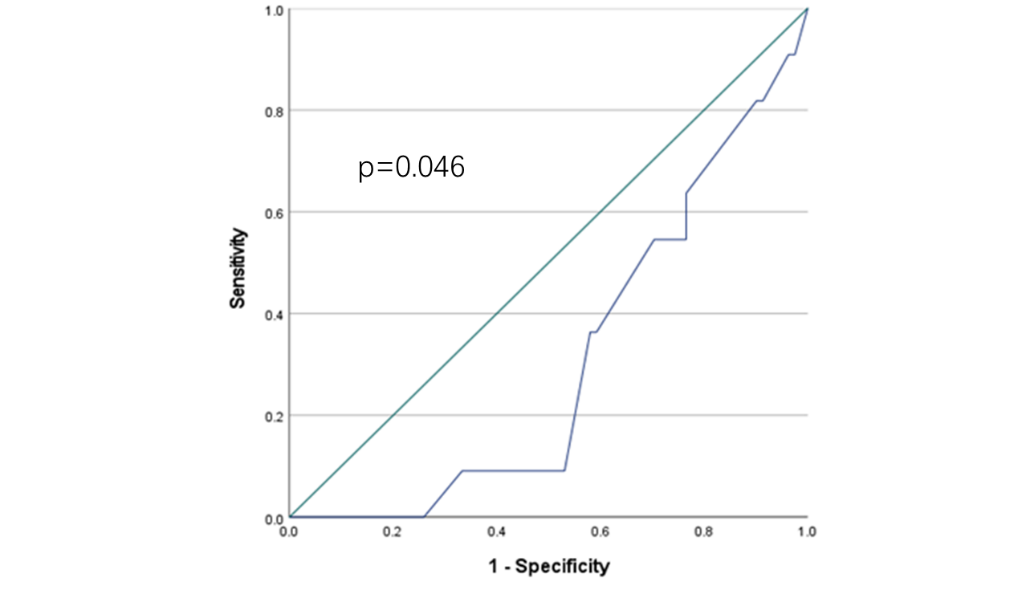


Supplementary Figure 2 The AUC of CPPRS for TRG in LAGC patients.


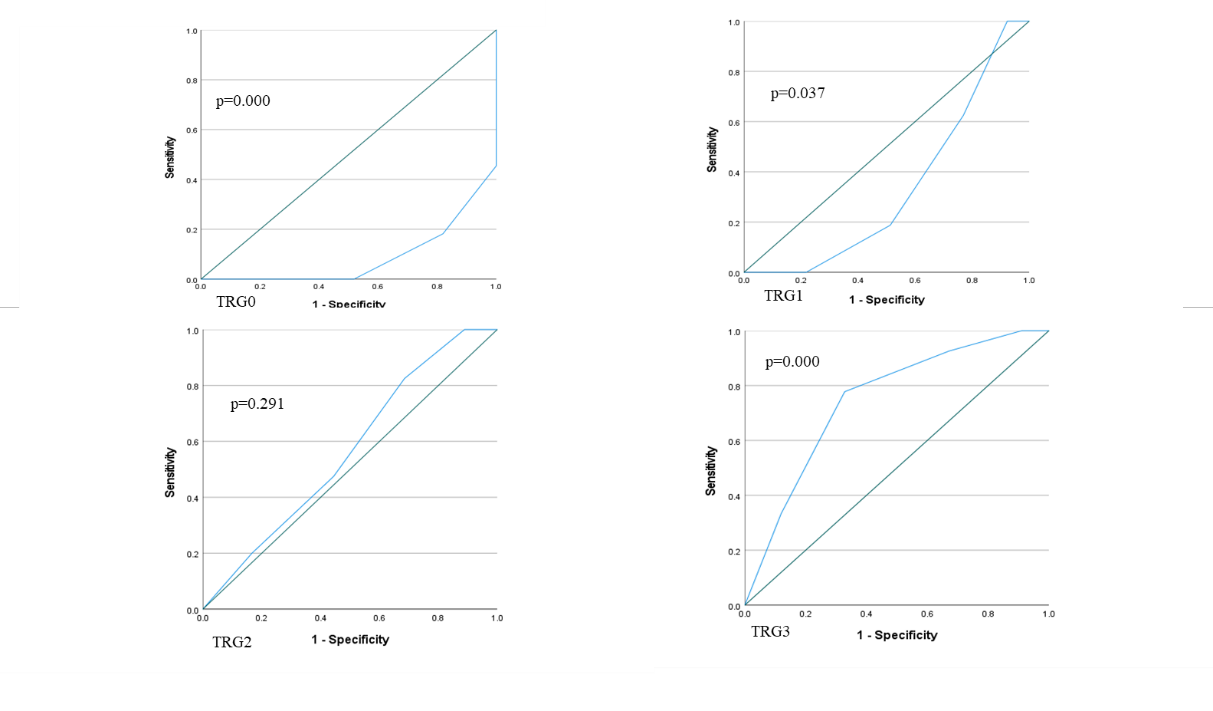

Supplement: Supplementary file 1 — Supplementary Material 1 [file 12957_2026_4373_MOESM1_ESM.docx]
